# New clinical application of high-intensity focused ultrasound: local control of synovial sarcoma

**DOI:** 10.1186/1477-7819-11-265

**Published:** 2013-10-08

**Authors:** Xiaoye Hu, Hongke Cai, Meiqi Zhou, Haifei He, Wei Tian, Yue Hu, Lirong Chen, Yongchuan Deng

**Affiliations:** 1 Department of Surgical Oncology, Second Affiliated Hospital, Zhejiang University College of Medicine, No. 88 Jiefang Road, Hangzhou, PR China; 2 Department of Pathology, Second Affiliated Hospital, Zhejiang University College of Medicine, No. 88 Jiefang Road, Hangzhou, PR China

**Keywords:** Cancer therapy, High-intensity focused ultrasound, Noninvasive surgery, Synovial sarcoma

## Abstract

High-intensity focused ultrasound (HIFU) is playing an increasingly important role in cancer therapy. Primary synovial sarcomas of the chest wall are extremely rare. We report the first case of noninvasive HIFU therapy for the control of synovial sarcoma. A 51-year-old man was diagnosed with spindle cell sarcoma on the left chest wall through lumpectomy. After four cycles of chemotherapy, local recurrence of the sarcoma was detected. Subsequent extended resection confirmed synovial sarcoma. After five cycles of a new chemotherapy option, the sarcoma relapsed again. Then the patient received five courses of HIFU; this completely ablated the sarcoma without complications. No chemotherapy, radiotherapy, or biological therapy has been applied since. Now the patient is stable and has a high quality of life.

## Background

Synovial sarcoma is a type of rare sarcoma in the soft tissue near the large joints of the arm or leg. Primary synovial sarcoma of the chest wall is extremely rare. The cytological features of the monophasic spindle cell and biphasic subtypes of synovial sarcoma, samples of which were obtained through fine needle aspiration, have been reported in several case studies [1]. Surgery is the mainstream therapy for synovial sarcoma. However, the post-surgery recurrence rate is still as high as 60% [2]. Moreover, synovial sarcoma is not sensitive to chemotherapy or radiotherapy [3]. So far, no report of high-intensity focused ultrasound (HIFU) therapy for synovial sarcoma has been found in the English literature. This report presents a case of synovial sarcoma of the left chest wall. Two surgical procedures and postoperative chemotherapy failed to control the tumor but HIFU therapy achieved good clinical efficacy.

## Case presentation

A solid texture mass, 2 cm in diameter, was found on the left chest wall of a 51-year-old man accompanied with local swelling and tenderness 1 year ago. Lumpectomy was performed in a local hospital. Postoperative pathological analysis confirmed the mass to be a spindle cell sarcoma. The patient was treated with a chemotherapy regimen of MTX 14.5 d1 + MTX 14.5 d8 + DDP 180 mg d15 + ADM 110 mg d17. After four cycles of chemotherapy, a similar mass with a more solid texture and some tenderness was found in the surgical site. Therefore, the patient was admitted to our hospital for a second surgery, expanded resection. Postoperative immunohistochemistry results indicated ER−, AR−, CK(Pan)±, CD34−, EMA−, CerbB2−, S-100−, Vimentin+++, EGFR−, P63−, SMA+, Calponin−, D2-40+, MBP−, CD99+++, β-catenin++, confirming diagnosis of synovial sarcoma. The patient received a new chemotherapy regimen of IFO 2 d1-5 + VP16 0.1 d1-5. However, follow-up magnetic resonance imaging (MRI) revealed that the tumor relapsed again after five cycles of chemotherapy. A new therapy plan was designed: HIFU. The HIFU procedure was performed using an FEP-BY02 HIFU system (Yuande Biomedical Engineering Co. Ltd, Beijing, China). A vertical scanning mode was chosen with a slice thickness of 2 mm. The ultrasonic transmitter worked at a frequency of 1.1 MHz and 11.0 MHz. GE LOGIQ 400CL was used for real-time monitoring during the therapy. The ultrasonic power was 130 W. Detailed therapeutic parameters were as follows: T_1_/T_2_ 990 ms/10 ms; 40 transmissions per therapeutic point with a distance of 2 mm between adjacent therapeutic points; treatment of each unit (five therapeutic points) for 200 seconds with an interval of 2 minutes between each unit; a spacing of 5 mm between adjacent treatment slices. The ablation effect was measured by MRI after the HIFU procedure.

**Figure 1:**
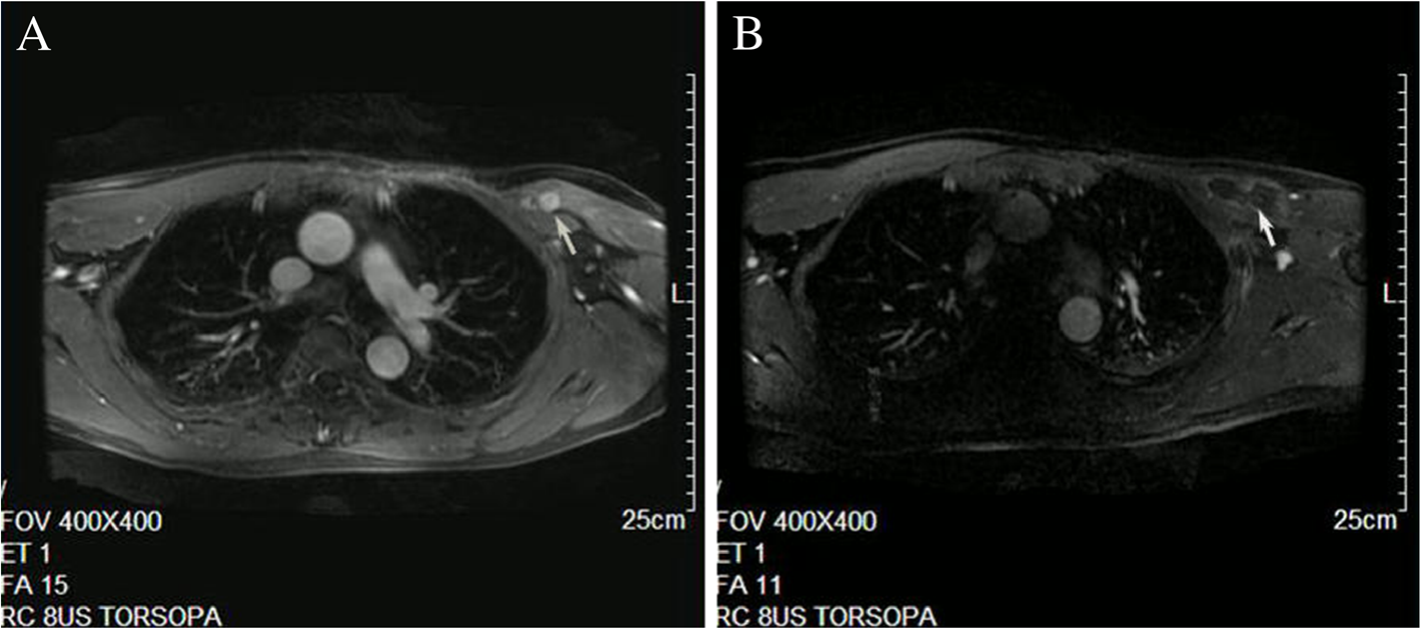
**Chest MRI. (A)** Before treatment, chest MRI shows enhanced tumor. **(B)** After treatment, arterial phase-enhanced MRI showed tumor was completely necrotized.

**Figure 2:**
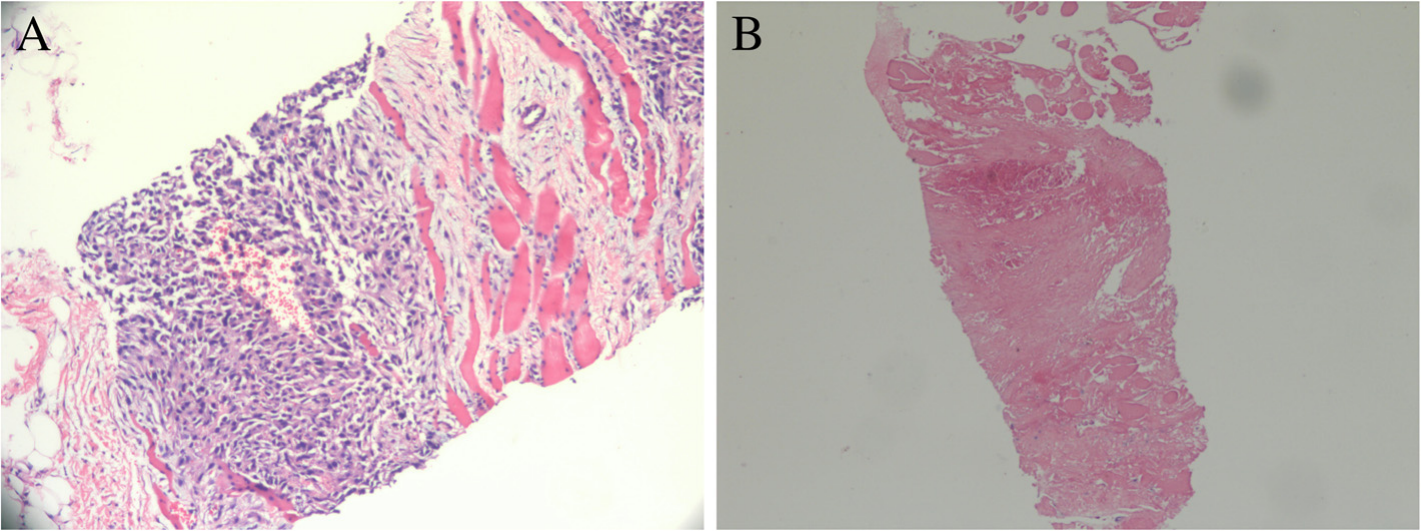
**H & E staining of samples obtained by needle aspiration. (A)** Before HIFU treatment, the sample contained a large number of synovial sarcoma cells and infiltration of the muscle layer. Magnification, ×200. **(B)** After treatment, tumor cells were not found, the sample only contained lymphocytes. Magnification, ×100.

After the first HIFU procedure, MRI demonstrated that the treated region was indicative of coagulation necrosis and homogeneous enhancement at the edge of the tumor. In total, five HIFU procedures were conducted, on 15 February 2011, 26 April 2011, 30 May 2011, 28 June 2011, and 6 September 2011. MRI conducted on 11 January 2011 and 7 September 2011 found complete coagulation necrosis in the therapeutic area (Figure 1A and B). Biopsy results before and after HIFU therapy are shown in Figure 2. Residual tumor cells were not found in repeated biopsy tests. HIFU completely ablated the tumor without complications and no further chemotherapy, radiotherapy or biological therapy was required for tumor control. The patient is now stable with a high quality of life. Further collection and analysis of follow-up data is underway.

## Discussion

Synovial sarcoma is characterized by local recurrence and difficulty in treatment. Surgery is the mainstream option for synovial sarcoma, accompanied by chemotherapy and radiotherapy. Further studies on molecular biology have revealed specific oncogene mutations and corresponding protection expression could serve as therapeutic targets [4]. Unfortunately, researchers have proved that chemotherapy and radiotherapy not only failed to improve progression-free survival or overall survival rate, but also led to poor life quality, owing to severe side effects [5]. With focused beams, HIFU can reach deep tissue and deliver localized warming, with temperatures reaching 70°C within 0.5 to 5 seconds [6]. HIFU could achieve coagulation necrosis through thermal effects, cavitation and mechanical effects without affecting normal tissues outside the therapeutic area [7]. HIFU has been widely used for prostate cancer [8], pancreatic cancer [9] and uterine fibroids [10]. Further in-depth research has focused on integration of HIFU therapy and chemotherapy or radiotherapy [11]. Currently, HIFU is being applied to treat a wider range of diseases: for example, Thomas Charrel [12] applied HIFU for the treatment of glaucoma. However, the application of HIFU is still at an exploratory stage. Many issues remain unsolved, such as the exact molecular biological effect of HIFU treatment, and how to improve therapeutic and dose efficiency. Nevertheless, HIFU represents an effective, repeatable and minimally invasive therapy option.

## Conclusions

This is the first report of effective noninvasive therapy for local control of synovial sarcoma. Without doubt, the development of HIFU will bring revolutionary technologies for cancer therapy.

## Consent

Written informed consent was obtained from the patient’s relatives for publication of this case report and accompanying images. A copy is available for review from the editor-in-chief of this journal.

## Abbreviations

H & E: Hematoxylin and eosin; HIFU: High-intensity focused ultrasound; MRI: Magnetic resonance imaging.

## Competing interests

The authors declare that they have no competing interests.

## Authors’ contributions

XH and HC collected the data and drafted the manuscript. MZ performed data analysis. HH, YH, and WT recruited the patient and collected study materials. YD designed the study and revised the manuscript. All authors read and approved the final manuscript.
